# Modeling opinion polarization on social media: Application to Covid-19 vaccination hesitancy in Italy

**DOI:** 10.1371/journal.pone.0291993

**Published:** 2023-10-02

**Authors:** Jonathan Franceschi, Lorenzo Pareschi, Elena Bellodi, Marco Gavanelli, Marco Bresadola

**Affiliations:** 1 Department of Mathematics “F. Casorati”, University of Pavia, Pavia, Italy; 2 Department of Mathematics and Computer Science, University of Ferrara, Ferrara, Italy; 3 Department of Engineering, University of Ferrara, Ferrara, Italy; 4 Department of Humanities, University of Ferrara, Ferrara, Italy; Roma Tre University: Universita degli Studi Roma Tre, ITALY

## Abstract

The SARS-CoV-2 pandemic reminded us how vaccination can be a divisive topic on which the public conversation is permeated by misleading claims, and thoughts tend to polarize, especially on online social networks. In this work, motivated by recent natural language processing techniques to systematically extract and quantify opinions from text messages, we present a differential framework for bivariate opinion formation dynamics that is coupled with a compartmental model for fake news dissemination. Thanks to a mean-field analysis we demonstrate that the resulting Fokker-Planck system permits to reproduce bimodal distributions of opinions as observed in polarization dynamics. The model is then applied to sentiment analysis data from social media platforms in Italy, in order to analyze the evolution of opinions about Covid-19 vaccination. We show through numerical simulations that the model is capable to describe correctly the formation of the bimodal opinion structure observed in the vaccine-hesitant dataset, which is witness of the known polarization effects that happen within closed online communities.

## 1 Introduction

Vaccination coverage, globally, has been at its highest levels for the last decades, with the notable exception of measles and diphtheria [1]. Measles outbreaks in particular [2] have raised concern in the Western public since they were about a disease that vaccination and treatments had reduced to a condition of rarity. The SARS-CoV-2 pandemic has been one compelling reason to rethink vaccination as an effective medical practice to prevent the spreading of diseases, especially in relation to the massive media coverage of the topic. The issue, in this case, is that a polarizing debate could exacerbate *vaccination hesitancy*, i.e., the reluctance in getting vaccinated (see [3–6], but also the recent [7–9]), with potentially dangerous implications for healthcare [10–13]. Moreover, unstable contexts like this one are more likely to develop irreparable fractures when misinformation is disseminated among people, and a positive reinforcement loop clusters the audience into isolated groups (the so-called *echo chambers*) where the only information shared is the one aligned with the majority point of view [5, 14–16]. Therefore, the need for the policymaker to be able to take informed decisions based on the understanding of the directions of the debate evolution is critical.

Vaccines are no stranger issue to mathematical literature, too [17, 18], especially the game-theoretical one. The *free-ride effect* of the portion of population not taking the possible risks associated with vaccination to enjoy the benefits given by the herd immunity are well known [19, 20]. So are opinions [21–25], especially in the context of multi-agent systems and kinetic theory more generally [26–32]. Within the same field, works have been proposed recently that borrow from the classical compartmental framework of epidemiological theory [33, 34], both for the spread of diseases and misinformation [35–39].

In this paper, we build on these elements to present a differential model, based on a mean-field description of agent dynamics, for the evolution of opinions in the presence of fake news spreaders. Although the model is mainly applied to the spread of fake news on social networks regarding the hesitancy to Covid-19 vaccination, its structure easily finds application in more general contexts where polarization of opinions within closed online communities is observed.

More in details, following the seminal paper [36], we consider a multi-agent population with a structure, where the modeling of fake news dissemination is managed via a set of compartments. In this setting, where the fake-news is treated as the spreading of a virus, the underlying variable that is shared by each agent characterizes a bivariate opinion distribution, that takes into account both positive and negative opinions about a given topic. This last aspect is crucial with a view to aligning the model with experimental data from *sentiment analysis* carried out on social media platforms, such as blogs and social networks. In such a situation, each opinion is inherently two-dimensional, as it classifies the polarity of a given text according to which level the opinion expressed is positive or negative [40–44].

We emphasize that, unlike [30, 36, 45], our starting point is a system of stochastic differential equations (SDEs) for the dynamics of opinions and not a binary interaction dynamics leading to a Boltzmann-type equation in the limit of a large number of agents. In fact, we are interested in modeling a situation in which agents interact simultaneously with the entire population, a scenario typical of group chats in instant messaging. As a consequence, the resulting model can be analyzed directly thanks to its mean-field approximation that permits to compute explicitly the steady states of the system without resorting to the quasi-invariant opinion approximation. The equilibrium states, in contrast with the classical case [30, 46], are characterized by a superposition of Beta distributions that give rise to bimodal shapes, i.e., individuals’ thoughts polarizing around different extreme positions, with a certain absence of compromise, in agreement with those observed opinion polarization effects in closed communities.

The model is then interfaced with available data concerning Covid-19 vaccination in Italy from the popular messaging app Telegram; one of its features is the possibility of having large online group chats focused on a topic of choice. They effectively form closed communities, where conversations experiment a low degree of noise: they are therefore ideal to analyze the evolution of sentiments about a certain subject. Numerical simulations show the model’s ability to interface correctly with the data extracted using NLP techniques and to describe the polarization phenomenon over time very well.

The rest of the manuscript is organized as follows: in Section 3, we present the stochastic differential model for opinion-formation processes characterized by two-dimensional vectors, which in the mean-field limit is approximated by a Fokker-Planck equation that allows us to compute steady states for marginals in explicitly solvable special cases. Next, in Section 4, we merge the model with a compartmental framework to take into account the potential spread of misinformation which can act as a catalyst for the polarization. In Section 5 we present the social media dataset and compare the evolution predicted by the model to the data one. Finally, in the last section some final considerations and concluding remarks are reported.

## 2 Materials and methods

All records from our datasets are posts from open, freely accessible, Telegram Italian chats, all of which are related to the topics of Covid-19 or vaccination:

- Io Non Mi Vaccino Chat
- Vittime vaccino Covid in Italia
- Mamme dissociate d’Italia/No vaccini COVID su bambini
- Singles italiani NON vaccinati
- COMBATTENTI NO BOOSTER—NO TERZA DOSE—NO VAC—NO GREEN PASS
- Personale Scuola—No Green Pass—No Booster Vax

We collected a total of 4077 posts from six different chats, from August 20th, 2021 to February 27th, 2022, which we anonymized and aggregated in order to obtain a unique, larger cluster. The collection method complied with the terms and conditions for the source of the data: in the Telegram Desktop version the “Export Chat history” functionality was used to download the data of interest from each chat in JSON format.

## 3 Mean-field models of bivariate opinion formation

When modelling the dynamics of opinions within individuals from a mathematical point of view, several approaches based on multi-agent interactions at various levels are possible [14, 26, 45, 47–50]. It is customary to set the interval [-1,1]⊆R as a natural space for the variable *w* representing the opinion, intending that radical positions are assumed as the absolute value |*w*| approaches 1, while neutral ones are assumed near 0. This choice embeds opinions as a continuous spectrum between positive and negative convictions and allows for a relatively simple description as a one-dimensional variable.

Here, we are setting ourselves in a subspace of the plane to better interpret the inherently two-dimensional nature of the description of opinions given by *natural language processing* (NLP) techniques like sentiment analysis, which assign scores based on how much a certain thought can be perceived as positive or negative, so that each record is associated with a pair of scores. Although a multivariate model for opinion requires greater care to devise it and to perform computations, it also gives us more coherent informations when aligning the model with data.

Of course, one can simplify the problem by projecting the two-dimensional opinion space [0, 1]^2^ onto a simpler one-dimensional space [−1, 1]. There are many ways to achieve this. Besides the previously mentioned mapping of positive and negative opinions in [0, 1] and [−1, 0], one can also achieve a one-dimensional mapping by considering the difference between positive and negative opinions in each output of the NLP algorithm. However, these one-dimensional embeddings, in addition to losing the correlation effects between positive and negative opinions, show limitations when it comes to polarization. For instance, if you choose to subtract the terms, a resulting neutral opinion close to zero is obtained both in the case of two bold contrasting statements and for a general mild thought.

The other main modeling choice is the use of SDEs instead of other alternatives, such as binary interactions described according to a broader interpretation of particle dynamics typical of statistical mechanics [30]. Here we are interested in modeling a situation in which agents interact simultaneously with the whole population at all times: this is the typical scenario of group chats within instant messaging applications.

### 3.1 A multi-agent stochastic differential model

**Fig 1:**
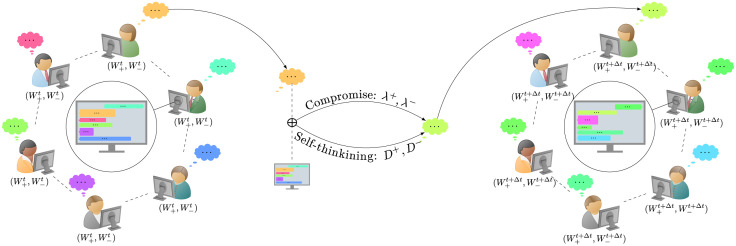
Opinions expressed by users in a group chat (represented here by different colors) are extrapolated by the messages via NLP and are denoted by a pair of continuous, time-dependent, real values $\left(W_{t}^{+},W_{t}^{-}\right)\in {\left[0,1\right]}^{2}$: One for how positive the opinion is and one for how negative it is, respectively. The dynamics is then characterized by the functions λ^+^, λ^−^ that define the compromise process and the functions *D*^+^, *D*^−^ that formalize individual self-thinking.

We consider a population of *N* indistinguishable agents: at the instant *t* ≥ 0, the *i*-th agent possesses an opinion expressed as a pair of sentiments (one positive and one negative), indicated by the vector ${\text{W}}_{t}^{i}=\left(W_{+,t}^{i},W_{-,t}^{i}\right)\in {\left[0,1\right]}^{2}$. The variables $W_{\pm ,t}^{t}$ can be thought as the intensities of those sentiments, so that when they are close to zero, they express a mild opinion, while when they are close to one they translates into fierce ones. In Fig 1 we sketch how the model works.

**Fig 2:**
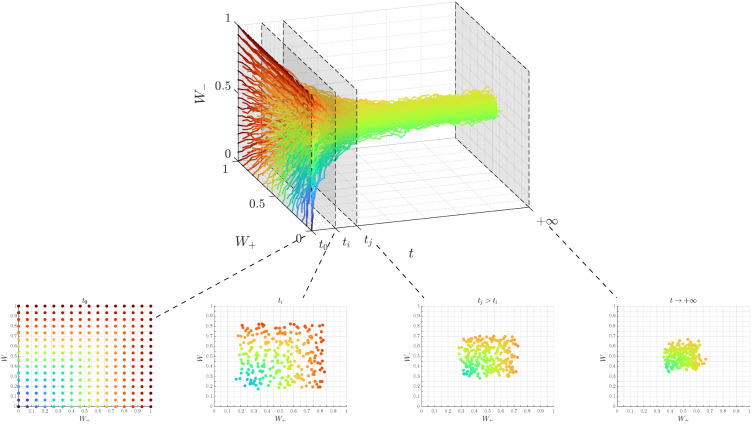
Simulation of model (1) using Euler-Maruyama scheme using *N* = 256 agents with λ^+^ = λ^−^ = *σ*_+_ = *σ*_−_ = 0.05. Here colors represent opinion’s intensity measured as ‖**W**_*t*_‖_∞_. Opinions initially are drawn from a uniform distribution and then, due to the compromise dynamics, concentrate toward the center of the unit square.

The continuous time evolution of the pair ${\text{W}}_{t}^{i}$ can be expressed via a stochastic differential system of the general form

$$\begin{array}{c} \left\{ \begin{array}{c} dW_{+,t}^{i}=\frac{1}{N}\sum_{j=1}^{N}\Lambda^{+}\left({\text{W}}_{t}^{i},{\text{W}}_{t}^{j}\right)\left(W_{+,t}^{j}-W_{+,t}^{i}\right)dt+\sigma_{+}D^{+}\left({\text{W}}_{t}^{i}\right)dB_{+,t}^{i}\\ dW_{-,t}^{i}=\frac{1}{N}\sum_{j=1}^{N}\Lambda^{-}\left({\text{W}}_{t}^{i},{\text{W}}_{t}^{j}\right)\left(W_{-,t}^{j}-W_{-,t}^{i}\right)dt+\sigma_{-}D^{-}\left({\text{W}}_{t}^{i}\right)dB_{-,t}^{i} \end{array}\right. \end{array}$$ (1)

where $\Lambda^{\pm }\left({\text{W}}_{t}^{i},{\text{W}}_{t}^{j}\right)$ are nonnegative functions characterizing the rate towards compromise when two agents *i* and *j* interact. Then *σ*_±_ are positive constant diffusion coefficients, while the nonnegative functions $D^{\pm }\left({\text{W}}_{t}^{i}\right)$ represent the local incidence of the diffusion effects due to self-thinking of agent *i*. The latter functions usually vanish at the boundary of [0, 1]^2^ so that people at extreme positions are less subject to noise effects. Finally, $dB_{\pm ,t}^{i}$ are independent one-dimensional Brownian motions to take into account the random nature of social interactions. In Fig 2 we show an example of simulation of model (1) for demonstration purposes.

It should be noted that the model introduced in (1), while inspired by the classical works by Hegselmann and Krause [51], Deffuant et al. [47], and Sznajd-Weron et al. [48], has some important differences due to the presence of a noise term in the interactions and the continuous-time description of the opinion formation process. In particular, due to the presence of noise, the dynamics may give rise to an inadmissible opinion vector. For this reason, the model is supplemented with appropriate boundary conditions that constrain the opinion vector within the domain [0, 1]^2^.

A typical example of compromise functions is represented by the bounded confidence model [51] where agents interact only if their opinions differ (component-wise) no more than a certain confidence level Δ_±_ ∈ [0, 1]

$$\Lambda^{\pm }\left({\text{W}}_{t}^{i},{\text{W}}_{t}^{j}\right)=\lambda^{\pm }\Psi (|W_{\pm ,t}^{i}-W_{\pm ,t}^{j}\left|\leq \Delta_{\pm }\right)$$

with Ψ(⋅) the indicator function and λ^±^ suitable positive constants. Further in this paper, we will restrict to the simplified situation where Δ_±_ = 1 (which means that agents can interact with everyone else) and thus $\Lambda^{\pm }\left({\text{W}}_{t}^{i},{\text{W}}_{t}^{j}\right)=\lambda^{\pm }$ represent the alignment strengths towards the current mean positive and negative opinions of the population

$$\begin{array}{c} M_{+,t}^{N}=\frac{1}{N}\sum_{j=1}^{N}W_{t,+}^{j},\;M_{-,t}^{N}=\frac{1}{N}\sum_{j=1}^{N}W_{t,-}^{j}. \end{array}$$ (2)

Next, we introduce the empirical measure

$$\begin{array}{c} f^{N}\left(\text{w},t\right)≔\frac{1}{N}\sum_{i=1}^{N}\delta \left(\text{w}-{\text{W}}_{t}^{i}\right), \end{array}$$ (3)

where **w** = (*w*_+_, *w*_−_) ∈ [0, 1]^2^ and *δ*(⋅) is the Dirac delta function, which counts how many agents share the same pair of opinions at time *t* ≥ 0. Our main goal is to analyze the evolution of the empirical measure of the system, especially when the number of individuals in the population grows large. The advantage of resorting to a limit procedure in order to study the mean-field version of system (1) is that under simplifying hypotheses it is possible to compute the stationary state of quantities of interest concerning the system.

### 3.2 Mean-field limit and Fokker-Planck approximation

A classical approach to formally analyze the behavior of the system when the number *N* of agents in the population is large, is to consider the *N*-particle probability density [52, 53]

$$f^{\left(N\right)}\left({\text{W}}_{t}^{1},\ldots ,{\text{W}}_{t}^{N},t\right)$$

and the associated first marginal

$$f_{1}^{\left(N\right)}\left({\text{W}}_{t}^{1},t\right)=\int_{{\left[0,1\right]}^{2N-2}}f^{\left(N\right)}\left({\text{W}}_{t}^{1},\ldots ,{\text{W}}_{t}^{N},t\right)\;d{\text{W}}_{t}^{2},\ldots ,d{\text{W}}_{t}^{N}.$$

and make the so-called *propagation of chaos assumption* on the marginals. More specifically, we assume that *f*^(*N*)^ ≈ *f*^⊗*N*^ for *N* ≫ 1, i.e., the random vectors ${\text{W}}_{t}^{1},\ldots ,{\text{W}}_{t}^{N}$ are approximately independently *f*(**w**, *t*)-distributed.

In this case, for *N* ≫ 1 we can write

$$\begin{array}{c} f^{N}\left(\text{w},t\right)\approx f\left(\text{w},t\right),\;\left(M_{+,t}^{N},M_{-,t}^{N}\right)\approx \left(m^{+}\left(t\right),m^{-}\left(t\right)\right)=\int_{{\left[0,1\right]}^{2}}f\left(\text{w},t\right)\text{w}\;d\text{w}, \end{array}$$ (4)

due to the law of large numbers. Consequently, the SDE model (1) becomes independent of *j* ≠ *i* and we obtain a so-called *Mc-Kean nonlinear process* (see, e.g., [54]) which, in the simplified situation where λ^±^ are non negative constants, reads

$$\begin{array}{c} \left\{ \begin{array}{c} dW_{t}^{+}=\lambda^{+}\left(m^{+}\left(t\right)-W_{t}^{+}\right)dt+\sigma_{+}D^{+}\left({\text{W}}_{t}\right)dB_{t}^{+},\\ dW_{t}^{-}=\lambda^{-}\left(m^{-}\left(t\right)-W_{t}^{-}\right)dt+\sigma_{-}D^{-}\left({\text{W}}_{t}\right)dB_{t}^{-}, \end{array}\right. \end{array}$$ (5)

with *f* ≔ law(**W**_*t*_), i.e., the measure induced by the process **W**_*t*_. The above system may be equivalently expressed by a nonlinear Fokker-Planck equation of the form [52, 53, 55]

$$\begin{array}{c} \begin{array}{cc} \frac{\partial }{\partial t}f\left(\text{w},t\right) & =\lambda^{+}\frac{\partial }{\partial w_{+}}\left[(w_{+}-m^{+}\left(t\right))f\left(\text{w},t\right)\right]+\lambda^{-}\frac{\partial }{\partial w_{-}}\left[(w_{-}-m^{-}\left(t\right))f\left(\text{w},t\right)\right]\\ & +\frac{\sigma_{+}^{2}}{2}\frac{\partial^{2}}{\partial w_{+}^{2}}(D^{+}{\left(\text{w}\right)}^{2}f\left(\text{w},t\right))+\frac{\sigma_{-}^{2}}{2}\frac{\partial^{2}}{\partial w_{-}^{2}}(D^{-}{\left(\text{w}\right)}^{2}f\left(\text{w},t\right)). \end{array} \end{array}$$ (6)

Eq (6) needs to be complemented with suitable no-flux boundary conditions that guarantee *f*(**w**, *t*) to be compactly supported in [0, 1]^2^

$$\begin{array}{c} \begin{array}{c} \lambda^{+}(w_{+}-m^{+}\left(t\right))f\left(\text{w},t\right)+\frac{\sigma_{+}^{2}}{2}\frac{\partial }{\partial w_{+}}(D^{+}{\left(\text{w}\right)}^{2}f\left(\text{w},t\right))=0,\;\text{on}\;\;w_{+}=0,1\\ \lambda^{-}(w_{-}-m^{-}\left(t\right))f\left(\text{w},t\right)+\frac{\sigma_{-}^{2}}{2}\frac{\partial }{\partial w_{-}}(D^{-}{\left(\text{w}\right)}^{2}f\left(\text{w},t\right))=0,\;\text{on}\;\;w_{-}=0,1. \end{array} \end{array}$$ (7)

Thanks to the above conditions we can introduce the normalization assumption

$$\int_{{\left[0,1\right]}^{2}}f\left(\text{w},t\right)d\text{w}=1,\;\forall \;t\geq 0.$$

We refer to [53], and the references therein, for rigorous results concerning the mean-field limit of stochastic particle system of type (1).

Note that, if in addition, at the boundary of [0, 1]^2^ we have

$$\begin{array}{c} D^{+}{\left(\text{w}\right)}^{2}f\left(\text{w},t\right)=D^{-}{\left(\text{w}\right)}^{2}f\left(\text{w},t\right)=0, \end{array}$$ (8)

integrating by parts and using the no-flux boundary conditions (7), we have conservation of the mean opinion

$$\frac{d\;\text{m}(t)}{dt}=\int_{{\left[0,1\right]}^{2}}\frac{\partial }{\partial t}f\left(\text{w},t\right)\text{w}\;d\text{w}=0.$$

If we now define the variances of the variables *w*^+^ and *w*^−^ as

$$V^{+}\left(t\right)=\int_{{\left[0,1\right]}^{2}}f\left(\text{w},t\right){\left(w^{+}-m^{+}\right)}^{2}\;d\text{w},\;V^{-}\left(t\right)=\int_{{\left[0,1\right]}^{2}}f\left(\text{w},t\right){\left(w^{-}-m^{-}\right)}^{2}\;d\text{w},$$

we have

$$\begin{array}{c} \frac{dV^{\pm }\left(t\right)}{dt}=-2\lambda^{\pm }V^{\pm }\left(t\right)+\sigma_{\pm }^{2}\int_{{\left[0,1\right]}^{2}}D^{\pm }{\left(\text{w}\right)}^{2}f\left(\text{w},t\right)\;d\text{w}. \end{array}$$ (9)

This shows that the particular choice of the functions *D*^±^(**w**) influences the behavior of the variance and so the convergence to equilibrium of the Fokker-Planck equation (6).

### 3.3 Equilibrium states for the marginal densities

The the functions *D*^±^(**w**) characterizing the local effect of diffusion, and thus the individual behavior of agents, turn out to be essential for the purpose of studying the equilibrium states of the system. For example taking

$$D^{\pm }=\left|w^{\pm }-m^{\pm }\right|,$$

where agents tends to reduce self-thinking as their opinion is close to the average, from (9) we get the uniform decay of the variances as soon as $2\lambda^{\pm }>\sigma_{\pm }^{2}$. In this case the long time behavior is characterized by a Dirac delta function *f*^∞^(**w**) = *δ*(**w** − **m**) where all agents are concentrated on the same opinion.

In the rest of the paper, we assume that opinions close to zero and one are less prone to random opinion effects, in the sense that both very moderate and more extreme individuals in expressing opinions have less freedom to change opinion, since they are already positioned in an extremal state. This assumption turns out to be essential in order to derive steady states in agreement with the experimental data, and differs from classical one-dimensional opinion models where individuals with an opinion around zero are assumed to be hesitant and so mostly prone to the effect of diffusion.

To this aim, we consider the local diffusion function to be such that *D*^+^(**w**) = *D*(*w*_+_) and *D*^−^(**w**) = *D*(*w*_−_) with

$$\begin{array}{c} D\left(w\right)=\sqrt{w(1-w)}, \end{array}$$ (10)

so that it vanishes in 0 and 1. This assumption, if the solution *f*(**w**, *t*) is sufficiently regular, guarantees conditions (8) at the boundary and therefore the mean opinion is independent from time. We refer to [30] for other admissible choices leading to interesting steady states. As we will see, thanks to (10) we are able to compute explicitly the steady state for the marginal densities.

Indeed, let us integrate directly system (6) with respect to the negative opinion *w*_−_, so that we have

$$\begin{array}{c} \begin{array}{cc} \frac{\partial }{\partial t}\int_{0}^{1}f\left(\text{w},t\right)\;dw_{-} & =\lambda^{+}\frac{\partial }{\partial w_{+}}[(w_{+}-m^{+})\int_{0}^{1}f\left(\text{w},t\right)\;dw_{-}]\\ & +{\lambda^{-}(w_{-}-m^{-})f\left(\text{w},t\right)|}_{0}^{1}\\ & +\frac{\sigma_{+}^{2}}{2}\frac{\partial^{2}}{\partial w_{+}^{2}}[w_{+}\left(1-w_{+}\right)\int_{0}^{1}f\left(\text{w},t\right)\;dw_{-}]\\ & +{\frac{\sigma_{-}^{2}}{2}\frac{\partial }{\partial w_{-}}[w_{-}\left(1-w_{-}\right)f\left(\text{w},t\right)]|}_{0}^{1}\;. \end{array} \end{array}$$ (11)

Thanks to the boundary conditions in (7) we have the simplification

$$\begin{array}{c} \frac{\partial }{\partial t}g\left(w_{+},t\right)=\lambda^{+}\frac{\partial }{\partial w_{+}}[(w_{+}-m^{+})g\left(w_{+},t\right)]+\frac{\sigma_{+}^{2}}{2}\frac{\partial^{2}}{\partial w_{+}^{2}}[w_{+}\left(1-w_{+}\right)g\left(w_{+},t\right)], \end{array}$$ (12)

where we denote the marginal density of the positive opinion as

$$g\left(w_{+},t\right)=\int_{0}^{1}f\left(\text{w},t\right)\;dw_{-}.$$

We can now compute the stationary solution *g*^∞^(*w*_+_) by observing that using the boundary conditions (7) it satisfies

$$\lambda^{+}[(w_{+}-m^{+})g^{\infty }\left(w_{+}\right)]+\frac{\sigma_{+}^{2}}{2}\frac{\partial }{\partial w_{+}}[w_{+}\left(1-w_{+}\right)g^{\infty }\left(w_{+}\right)]=0.$$

**Fig 3:**
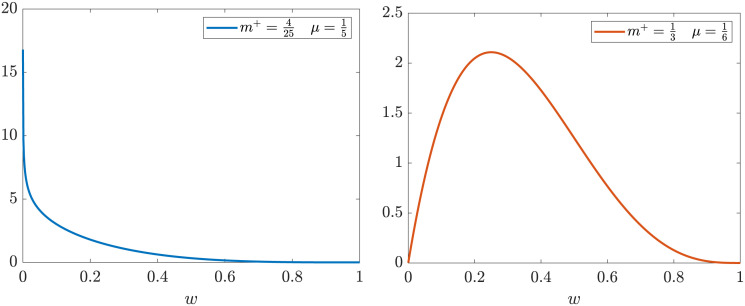
Examples of stationary marginal opinion distributions *g*^∞^(*w*_+_) characterized by Beta(*w*_+_; *a*, *b*) functions obtained with different choices of *m*^+^ and *μ*. Left: *m*^+^ = 4/25, *μ* = 1/5, corresponding to *a* = 0.8, *b* = 4.2. We can see that the function tends to infinity as we approach the left boundary. Right: *m*^+^ = 1/3, *μ* = 1/6, corresponding to *a* = 2, *b* = 4, with a unimodal structure.

Thus, *g*^∞^ is computed explicitly as [30]

$$\begin{array}{c} g^{\infty }\left(w_{+}\right)=C_{+}w_{+}^{m^{+}/\mu_{+}-1}{\left(1-w_{+}\right)}^{\left(1-m^{+}\right)/\mu_{+}-1}, \end{array}$$ (13)

where $\mu_{+}≔\lambda^{+}/\sigma_{+}^{2}$, *m*_+_ ∈ (0, 1) and *C*_+_ is a normalization constant which depends on all parameters appearing in (13). Eq (13) represents a Beta distribution of the form Beta(*w*; *a*, *b*) with *a* = *m*^+^/*μ*_+_ and *b* = (1 − *m*^+^)/*μ*_+_ (see Fig 3). Note also that since *a*, *b* > 0 condition (8) is always guaranteed.

Analogous calculations can be performed to obtain a closed expression for the steady state of the marginal density of the negative opinion *h*(*w*_−_, *t*) which reads

$$\begin{array}{c} h^{\infty }\left(w_{-}\right)=C_{-}w_{-}^{m^{-}/\mu_{-}-1}{\left(1-w_{-}\right)}^{\left(1-m^{-}\right)/\mu_{-}-1}, \end{array}$$ (14)

where now $\mu_{-}≔\lambda^{-}/\sigma_{-}^{2}$, *m*_−_ ∈ (0, 1) and *C*_−_ is a normalization constant. In Fig 3 are reported examples of various stationary marginal opinion distributions.

One of their interesting properties is that they are flexible enough to give rise to several different shapes, including some that are unbounded near the ends of the support. This is representative of extreme polarization phenomena in which the vast majority of the population shares extreme ideas. Beta distributions are also *unimodal*, i.e., agents well described by a Beta tend to aggregate around a certain, unique, value, where this tendency depends on the variance of the distribution. However, this implies that a similar model would not be able to accurately describe different kinds of polarization, the ones that are local to certain population subsets and that may have a *bimodal* structure.

These latter phenomena are the ones more commonly associated with the formation of separate clusters within communities after the population has been exposed to fake news: misinformation exacerbates underlying radical opinions in a group of individuals, who then progressively proceed to discard any other belief. While this happens broadly, at different levels, (the phenomenon of *echo chambers*), the less moderate one are often the one that raise more concern [56–61].

Therefore, the model should also take into account the effects brought by the dissemination of fake news that also lead to changes of the average opinions within subgroups of individuals. This will be explored in the next section.

## 4 Merging opinion formation with fake news dissemination

The full model focuses again on a structured population where *N* indistinguishable agents all share a vector-valued variable **w** ∈ [0, 1]^2^. In the current setting, concurrent to the opinion formation process is also spread of misinformation, whose dynamics can be fruitfully approached through the compartmental framework typical of epidemiology [33, 34, 36].

### 4.1 Defining fake news

**Fig 4:**
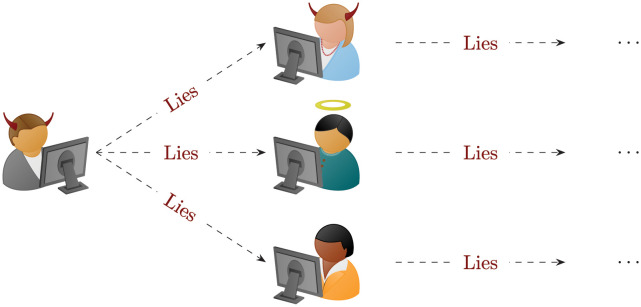
Defining fake news: here the evil look of the person on the left symbolizes the purpose of voluntarily mislead others with false information; whereas the first recipients of the news spread it, in turn, either animated by the same will (evil agent, top), by the desire of sharing helpful or otherwise legitimate information (angelic agent, middle) or finally guided by no specific goal (neutral agent, bottom).

Defining what fake news is and why it is a phenomenon deserving its own category (think for instance to other classifications of lies, e.g., scams, hoaxes, urban legends and etc.) is itself challenging. Our approach will be to consider fake news any piece of information whose *initial* diffusion is made with the purpose of mislead people intentionally. The word ‘initial’ here is key, because fake news is most often spread by people who do not know (or care) it is false (see Fig 4). This has been linked to the concept of *post-truth* and explored also from a philosophical point of view [62]; see [36] and the references therein for a recent overview on the different approaches for fake news detection.

### 4.2 A compartmental model for fake news spreading

For what concerns the dissemination of fake news, we assume that the people within the community can be divided into four disjoint compartments: susceptible (or ignorant), exposed (or incubator), infected (or spreader) and recovered (or stifler). Concerning the nomenclature, we opted for the epidemiological convention here to adhere to the previous works [36, 37]. We refer to [36] and the references therein for other popular choices.

Then we use suitable differential equations to describe the way individuals change compartment. To each compartment will be assigned its initial as identifying letter, so that we will refer to them as the set C≔{S,E,I,R}. We shall therefore study the evolution of the opinion’s distribution of the agents in each compartment, noted, respectively, by *f*_*S*_ = *f*_*S*_(**w**, *t*), *f*_*E*_ = *f*_*E*_(**w**, *t*), *f*_*I*_ = *f*_*I*_(**w**, *t*), and *f*_*R*_ = *f*_*R*_(**w**, *t*).

Like before, we restrict ourselves to consider a reduced time-span, during which we can assume that the population is fixed, i.e., nobody enters or leaves it; This choice is based on the average lifespan of fake news. Thus, we set the overall opinion distribution as a probability density for all *t* ≥ 0, i.e.,

∫[0,1]∑J∈CfJ(w,t)dw=1,t>0.

The quantities in the first column of

gJ(w+,t)=∫[0,1]fJ(w,t)dw-mJ+(t)=1ρJ(t)∫[0,1]2w-fJ(w,t)dw,hJ(w-,t)=∫[0,1]fJ(w,t)dw+mJ-(t)=1ρJ(t)∫[0,1]2w+fJ(w,t)dw

denote the marginals densities, i.e., the fractions of the population that belongs to compartment J∈C with positive and negative opinion, respectively, at time *t* ≥ 0, while in the second column we denote the mean relative to the positive and to the negative opinion, respectively. Finally,

$$\begin{array}{c} \rho_{J}\left(t\right)=\int_{{\left[0,1\right]}^{2}}f_{J}\left(\text{w},t\right)\;d\text{w} \end{array}$$

is the total mass fraction of agents in the compartment *J*.

**Fig 5:**
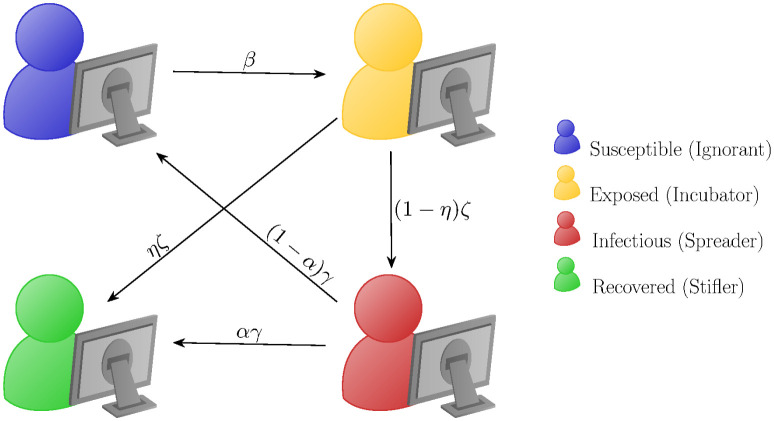
Compartmental dynamic and parameters in the fake news SEIR model (15): People get exposed to fake news with a contact rate *β* with infected individuals, after a latency period 1/*ζ* with probability 1 − *η* they start spreading it until they finally stop after an average time 1/*γ* and become ‘immunized’ or uninterested in it, thus removing themselves from the dissemination dynamics with probability *α*.

**Fig 6:**
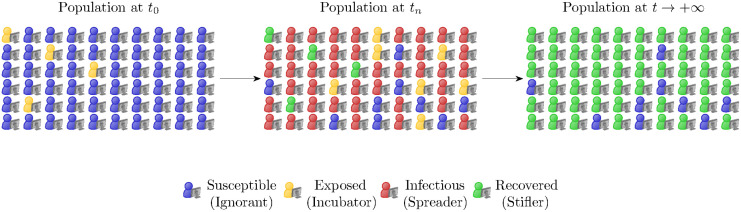
Evolution in time of the dissemination of fake news within a population for model (15). For large times the fake-news infection disappears and the population is composed only by susceptible and recovered individuals.

When the fake-news dynamic is independent from the opinion of individuals it follows the simple system of ordinary differential equations [36]

$$\begin{array}{c} \left\{ \begin{array}{cc} \frac{d\rho_{S}\left(t\right)}{dt} & =-\beta \rho_{S}\left(t\right)\rho_{I}\left(t\right)+\left(1-\alpha \right)\gamma \rho_{I}\left(t\right)\\ \frac{d\rho_{E}\left(t\right)}{dt} & =\beta \rho_{S}\left(t\right)\rho_{I}\left(t\right)-\zeta \rho_{E}\left(t\right)\\ \frac{d\rho_{I}\left(t\right)}{dt} & =\left(1-\eta \right)\zeta \rho_{E}\left(t\right)-\gamma \rho_{I}\left(t\right)\\ \frac{d\rho_{R}\left(t\right)}{dt} & =\eta \zeta \rho_{E}\left(t\right)+\alpha \gamma \rho_{I}\left(t\right) \end{array}\right. \end{array}$$ (15)

with *ρ*_*S*_(*t*) + *ρ*_*E*_(*t*) + *ρ*_*I*_(*t*) + *ρ*_*R*_(*t*) = 1. We will refer to it as a SEIR model for fake-news spreading. Basically, susceptible agents get exposed at a rate that is proportional to the probability of them interacting with an active spreader (i.e., an infectious individual). Once they are exposed, they wait for an average time 1/*ζ* and start disseminate the fake news with probability 1 − *η*. After an average time 1/*γ*, they stop doing so and are removed permanently from the dynamics with probability *α*. A schematic depiction of the dynamics is showed in Fig 5, whereas in Fig 6 an example of evolution within a closed population is sketched. For simplicity, we assume *η* = 0, *α* = 1 so that the exposed individuals, after the latency period, always start to spread the fake-news and after an average time spreaders are permanently removed from the dynamic.

If we combine the dissemination dynamics with the opinion formation process described in the previous section we obtain the following mean-field model

$$\begin{array}{cc} \frac{\partial f_{S}\left(\text{w},t\right)}{\partial t} & =-K\left(f_{S},f_{I}\right)\left(\text{w},t\right)+\lambda_{S}^{+}\frac{\partial }{\partial w_{+}}\left[(w_{+}-m_{+}\left(t\right))f_{S}\left(\text{w},t\right)\right]\\ & +\lambda_{S}^{-}\frac{\partial }{\partial w_{-}}\left[(w_{-}-m_{-}\left(t\right))f_{S}\left(\text{w},t\right)\right]\\ & +\frac{\sigma_{+,S}^{2}}{2}\frac{\partial^{2}}{\partial w_{+}^{2}}(D{\left(w_{+}\right)}^{2}f_{S}\left(\text{w},t\right))+\frac{\sigma_{-,S}^{2}}{2}\frac{\partial^{2}}{\partial w_{-}^{2}}(D{\left(w_{-}\right)}^{2}f_{S}\left(\text{w},t\right)), \end{array}$$ (16)

$$\begin{array}{cc} \frac{\partial f_{E}\left(\text{w},t\right)}{\partial t} & =K\left(f_{S},f_{I}\right)\left(\text{w},t\right)-\zeta \left(\text{w}\right)f_{E}\left(\text{w},t\right)+\lambda_{E}^{+}\frac{\partial }{\partial w_{+}}\left[(w_{+}-m_{+}\left(t\right))f_{E}\left(\text{w},t\right)\right]\\ & +\lambda_{E}^{-}\frac{\partial }{\partial w_{-}}\left[(w_{-}-m_{-}\left(t\right))f_{E}\left(\text{w},t\right)\right]+\frac{\sigma_{+,E}^{2}}{2}\frac{\partial^{2}}{\partial w_{+}^{2}}(D{\left(w_{+}\right)}^{2}f_{E}\left(\text{w},t\right))\\ & +\frac{\sigma_{-,E}^{2}}{2}\frac{\partial^{2}}{\partial w_{-}^{2}}(D{\left(w_{-}\right)}^{2}f_{E}\left(\text{w},t\right)), \end{array}$$ (17)

$$\begin{array}{cc} \frac{\partial f_{I}\left(\text{w},t\right)}{\partial t} & =\zeta \left(\text{w}\right)f_{E}\left(\text{w},t\right)-\gamma \left(\text{w}\right)f_{I}\left(\text{w},t\right)+\lambda_{I}^{+}\frac{\partial }{\partial w_{+}}\left[(w_{+}-m_{+}\left(t\right))f_{I}\left(\text{w},t\right)\right]\\ & +\lambda_{I}^{-}\frac{\partial }{\partial w_{-}}\left[(w_{-}-m_{-}\left(t\right))f_{I}\left(\text{w},t\right)\right]+\frac{\sigma_{+,I}^{2}}{2}\frac{\partial^{2}}{\partial w_{+}^{2}}(D{\left(w_{+}\right)}^{2}f_{I}\left(\text{w},t\right))\\ & +\frac{\sigma_{-,I}^{2}}{2}\frac{\partial^{2}}{\partial w_{-}^{2}}(D{\left(w_{-}\right)}^{2}f_{I}\left(\text{w},t\right)), \end{array}$$ (18)

$$\begin{array}{cc} \frac{\partial f_{R}\left(\text{w},t\right)}{\partial t} & =\gamma \left(\text{w}\right)f_{I}\left(\text{w},t\right)+\lambda_{R}^{+}\frac{\partial }{\partial w_{+}}\left[(w_{+}-m_{+}\left(t\right))f_{R}\left(\text{w},t\right)\right]\\ & +\lambda_{R}^{-}\frac{\partial }{\partial w_{-}}\left[(w_{-}-m_{-}\left(t\right))f_{R}\left(\text{w},t\right)\right]\\ & +\frac{\sigma_{+,R}^{2}}{2}\frac{\partial^{2}}{\partial w_{+}^{2}}(D{\left(w_{+}\right)}^{2}f_{R}\left(\text{w},t\right))+\frac{\sigma_{-,R}^{2}}{2}\frac{\partial^{2}}{\partial w_{-}^{2}}(D{\left(w_{-}\right)}^{2}f_{R}\left(\text{w},t\right)), \end{array}$$ (19)

where

$$\begin{array}{c} m_{+}=\sum_{J\in C}\rho_{J}m_{J}^{+},\;m_{-}=\sum_{J\in C}\rho_{J}m_{J}^{-}. \end{array}$$ (20)

The functional

K(fS,fI)(w,t)=fS(w,t)∫[0,1]2κ(w*)fI(w*,t)dw* (21)

is the local incidence rate of interactions between susceptible and infectious individuals, where *κ*(**w**) is a contact function which measures the impact of the opinion in the dissemination of fake-news. A simplifying assumption is that *κ*(⋅, ⋅) is separable in the two variables, i.e., $\kappa \left(\text{w}\right)=\beta k\left(w_{+}\right)\bar{k}\left(w_{-}\right)$, with *β* > 0 a constant. That way, we can think the impact of the strength of positive sentiment acts independently by the one of the strengths of the negative sentiment and we can decouple their evolution. A choice of particular interest would be one in which *κ*(⋅) is a function of the sole variable *w*_+_ (respectively, *w*_−_).

System (16)–(19) needs to be complemented by the no flux boundary conditions for all J∈C

$$\begin{array}{c} \lambda_{J}^{+}(w_{+}-m_{+}\left(t\right))f_{J}\left(\text{w},t\right)+\frac{\sigma_{+,J}^{2}}{2}\frac{\partial }{\partial w_{+}}(D{\left(w_{+}\right)}^{2}f_{J}\left(\text{w},t\right))=0,\;\text{on}\;\;w_{+}=0,1,\\ \lambda_{J}^{-}(w_{-}-m_{-}\left(t\right))f_{J}\left(\text{w},t\right)+,\frac{\sigma_{-,J}^{2}}{2}\frac{\partial }{\partial w_{-}}(D{\left(w_{-}\right)}^{2}f_{J}\left(\text{w},t\right))=0,\;\text{on}\;\;w_{-}=0,1. \end{array}$$ (22)

Note that, if the alignment rates $\lambda_{J}^{\pm }=\lambda^{\pm }$ independent from J∈C, as a consequence of the above boundary conditions and the choice of the diffusion function (10), the quantities *m*_+_(*t*) and *m*_−_(*t*) are conserved in time.

### 4.3 Stationary marginal densities

If in system (16)–(19) we choose a constant function *κ*(⋅, ⋅) ≡ *β* > 0 as well as constant epidemiological parameters we obtain again system (15) with *α* = *η* = 1 by integrating in the variable **w**. As a consequence, classical results in epidemiology [33] guarantee that when *t* → ∞ the fake-news spreading vanishes and we have both *ρ*_*E*_(*t*) → 0 and *ρ*_*I*_(*t*) → 0. Moreover, $\rho_{S}\left(t\right)\to \rho_{S}^{\infty }$ and $\rho_{R}\left(t\right)\to \rho_{R}^{\infty }=1-\rho_{S}^{\infty }$ where $\rho_{S}^{\infty }$ solves

$$\begin{array}{c} \text{log}\;(\frac{\rho_{S}}{\rho_{S}\left(0\right)})=\frac{\beta }{\gamma }\left(1-\rho_{S}^{\infty }\right). \end{array}$$ (23)

Let us denote with $m_{+}^{\infty }$, $m_{-}^{\infty }$ the large time behavior Similarly the evolutions of the first moments are obtained by integrating in **w** after multiplication for *w*_±_ to obtain

$$\begin{array}{c} \left\{ \begin{array}{c} \frac{d}{dt}\left(\rho_{S}\left(t\right)m_{S}^{\pm }\left(t\right)\right)=-\beta \rho_{S}\left(t\right)m_{S}^{\pm }\left(t\right)\rho_{I}\left(t\right)-\lambda_{S}^{\pm }\rho_{S}\left(m_{S}^{\pm }-m_{\pm }\right)\\ \frac{d}{dt}\left(\rho_{E}\left(t\right)m_{E}^{\pm }\left(t\right)\right)=\beta \rho_{S}\left(t\right)m_{S}^{\pm }\left(t\right)\rho_{I}\left(t\right)-\zeta \rho_{E}\left(t\right)-\lambda_{E}^{\pm }\rho_{E}\left(m_{E}^{\pm }-m_{\pm }\right)\\ \frac{d}{dt}\left(\rho_{I}\left(t\right)m_{I}^{\pm }\left(t\right)\right)=\zeta \rho_{E}\left(t\right)m_{E}^{\pm }\left(t\right)-\gamma \rho_{I}\left(t\right)m_{I}^{\pm }\left(t\right)-\lambda_{I}^{\pm }\rho_{I}\left(m_{I}^{\pm }-m_{\pm }\right)\\ \frac{d}{dt}\left(\rho_{R}\left(t\right)m_{R}^{\pm }\left(t\right)\right)=\gamma \rho_{I}\left(t\right)m_{I}^{\pm }\left(t\right)-\lambda_{R}^{\pm }\rho_{R}\left(m_{R}^{\pm }-m_{\pm }\right). \end{array}\right. \end{array}$$ (24)

So that for large times we have $m_{\pm }\left(t\right)\to m_{\pm }^{\infty }$ and $m_{S}^{\pm }=m_{R}^{\pm }=m_{\pm }^{\infty }$.

Then, if we integrate (16)–(19) with respect to *w*_−_ in the same way we did for equation (6), using the boundary conditions (22) we have that at the stationary state the marginals for the positive opinion satisfy

$$\begin{array}{cc} \frac{\partial }{\partial w_{+}}[(\lambda_{S}^{+}w_{+}-m_{+}^{\infty })g_{S}^{\infty }\left(w_{+}\right)]+\frac{\sigma_{+,S}^{2}}{2}\frac{\partial^{2}}{\partial w_{+}^{2}}[w_{+}\left(1-w_{+}\right)g_{S}^{\infty }\left(w_{+}\right)] & =0,\\ \frac{\partial }{\partial w_{+}}[(\lambda_{R}^{+}w_{+}-m_{+}^{\infty })g_{R}^{\infty }\left(w_{+}\right)]+\frac{\sigma_{+,R}^{2}}{2}\frac{\partial^{2}}{\partial w_{+}^{2}}[w_{+}\left(1-w_{+}\right)g_{R}^{\infty }\left(w_{+}\right)] & =0, \end{array}$$

which provide the stationary distributions

$$\begin{array}{c} \begin{array}{cc} g_{S}^{\infty }\left(w_{+}\right) & =\rho_{S}^{\infty }C_{S}^{+}w_{+}^{m_{+}^{\infty }/\mu_{S}^{+}-1}{\left(1-w_{+}\right)}^{\left(1-m_{+}^{\infty }\right)/\mu_{S}^{+}-1},\\ g_{R}^{\infty }\left(w_{+}\right) & =\left(1-\rho_{S}^{\infty }\right)C_{R}^{+}w_{+}^{m_{+}^{\infty }/\mu_{R}^{+}-1}{\left(1-w_{+}\right)}^{\left(1-m_{+}^{\infty }\right)/\mu_{R}^{+}-1} \end{array} \end{array}$$ (25)

where $\mu_{S}^{+}=\lambda_{S}^{+}/\sigma_{+,S}^{2}$, $\mu_{R}^{+}=\lambda_{R}^{+}/\sigma_{+,R}^{2}$ and $C_{S}^{+}$, $C_{R}^{+}$ are normalization constants.

Introducing analogous hypotheses, we obtain the same result for the total marginal density of negative opinions

$$\begin{array}{c} \begin{array}{cc} h_{S}^{\infty }\left(w_{-}\right) & =\rho_{S}^{\infty }C_{S}^{-}w_{-}^{m_{-}^{\infty }/\mu_{S}^{-}-1}{\left(1-w_{-}\right)}^{\left(1-m_{-}^{\infty }\right)/\mu_{S}^{-}-1},\\ h_{R}^{\infty }\left(w_{-}\right) & =\left(1-\rho_{S}^{\infty }\right)C_{R}^{-}w_{-}^{m_{-}^{\infty }/\mu_{R}^{-}-1}{\left(1-w_{-}\right)}^{\left(1-m_{-}^{\infty }\right)/\mu_{R}^{-}-1} \end{array} \end{array}$$ (26)

with $\mu_{S}^{-}=\lambda_{S}^{-}/\sigma_{-,S}^{2}$, $\mu_{R}^{-}=\lambda_{R}^{-}/\sigma_{-,R}^{2}$ and $C_{S}^{-}$, $C_{R}^{-}$ normalization constants.

**Fig 7:**
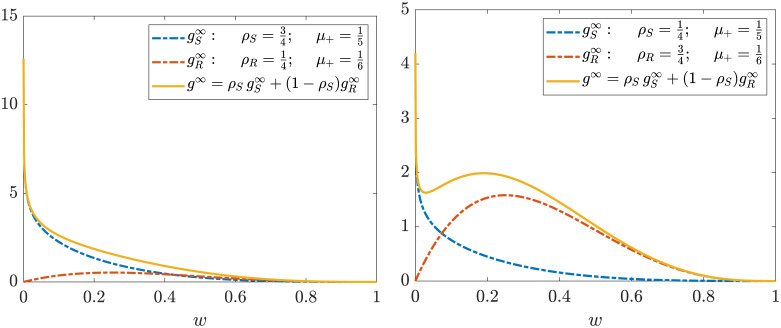
Examples of stationary solutions obtained from (25) with different choices of parameters *ρ*_*S*_, *ρ*_*R*_, *m*_+_, $\lambda_{S}^{+}$, $\lambda_{R}^{+}$, *σ*_+,*S*_ and *σ*_−,*S*_. We can see that the resulting function can have both a unimodal character (left) or a bimodal behavior (right).

This means that, depending on the given regime of parameters, since the total marginal density for the positive or negative opinions is the mixture of two Beta distributions, it can be unimodal or bimodal (see Fig 7).

## 5 Application to Covid-19 vaccination hesitancy

As we mentioned in the introduction, the proposed model is suitable to describe the evolution of opinions within closed online communities where data are collected through a suitable use of NLP techniques like sentiment analysis. Here, we present the particular case of selected groups of people which shared preoccupation for the Italian vaccination campaign in response to the SARS-CoV-2 pandemic [63]. More in details, they all share, to a certain extent, *vaccination hesitancy*.

### 5.1 Data collection

We comment here about the data records that we outlined in the Methods’ Section. All chat posts focus on vaccination, from various perspectives and scopes, which refer to different social groups the users belong to.

References to conspiracy theories, plain misinformation, rage bursts and mockery, all mix with sincere pleas of explanations on vaccination as well as other measures employed by the former Italian government to combat and contain the effect of the SARS-CoV-2 pandemic on both the national healthcare and economy.

Using techniques proper of the framework of *sentiment analysis* [40–44], we assigned to each post a pair $\left({\bar{w}}_{+},{\bar{w}}_{-}\right)\in {\left[0,1\right]}^{2}$ of scores, respectively positive and negative which reflect how good or bad the opinion of the user might be in that instant of time.

Scores are assigned in an automatic fashion by SentIta [44] a pre-trained model which analyzes the content of each post. SentIta is a Bidirectional LSTM-Convolutional Neural Network with two output signals ranging between 0 and 1, which performs sentiment analysis on Italian texts (no translation was required). The model is released as an open-source Python library (https://nicgian.github.io/Sentita/, last accessed on June 1, 2023), which we directly applied to our dataset. The model has been trained and tested on Sentipolc2016 [64] and ABSITA2018 [65] datasets for a total of 15,000 positively and negatively labelled Italian sentences plus 90,000 Wikipedia sentences automatically labelled as neutral. The following is a post from our dataset which was evaluated as a score pair of (0.055934787, 0.820981), with the English translation aside.

Ma tanto anche se a queste persone diciamo non fatevi più altre dosi che vi fanno male, non ci ascoltano. Ormai per loro siamo noi i cattivi, e non quelli che veramente sono i cattivi. Ormai non hanno in mente altro. Pensano solo alla prossima dose e ai cattivi no vax.

Still even if we tell this people don’t take any more doses, that they hurt you, they won’t listen. At this point to them it’s us the bad guys and not the ones that are the bad guys for real. By now they have nothing else in mind. They just think about the next dose and to the bad no-vaxs.

The following one instead scored a pair (0.31506833, 0.69410014).

Io di Paragone non mi fido… cmq io la mia battaglia la faccio qui. E siamo davvero na marea. Non so come e in Italia ma qui pian piano la gente si sta svegliando, anche i vaccinati si stanno unendo a noi.

I don’t trust Paragone [former Italian politician, authors’ note]… however I fight my battle here. And we really are a ton. I don’t know how and in Italy but here slowly but surely people are waking up, even the vaccinated are joining us.

As a last example, we report one of the few posts that were originally written in English (score of (0.028655171, 0.045119375)).

**truth revealed**: FAUCI just confess on a live stream with Mark Zuckerberg that vaccination actually may cause the problem.

The last post is revealing of some of the issues involved in using software-based sentiment analysis techniques: the form may be neutral and plain, but its content arguably is. Also the small caps text is typical of the sensationalist tones affine with conspiracy theories and fake news in general.

In spite of sentiment analysis having become spread both in academic and corporate works [43, 66, 67], its evaluation is not free from risk: since NLP is a relatively young discipline which faces lots of challenging tasks, there are no current one-solves-all approaches for parsing human-produced syntax in a robust way. Besides, online chats might not be the most suited environment for unambiguous, error-free communication, not to mention the use of non-verbal means, such as non-plain-text characters (emojis, for instance) to express emotions and concepts which necessarily would go undetected by a not instructed software. Hence, the evaluation of the records in our datasets comes with inherent uncertainties. Here we do not try to quantify these uncertainties, we refer to [68] for related approaches to uncertain data in compartmental models.

In the following, when interfacing data with our model, we always considered aggregate data, i.e., scores gathered for posts from every group chat combined into a unique dataset. Moreover, if not otherwise specified, we always discretized the dataset into a grid of 20 × 20 bins.

**Fig 8:**
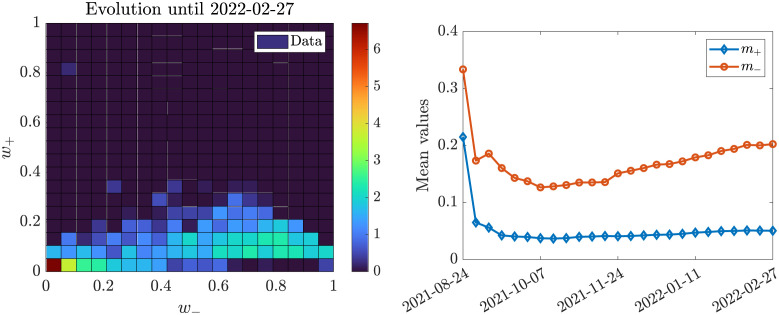
Left: final time snapshot of the dataset. The base 2-logarithm of the data is used for coloring in order to better appreciate the differences in magnitude. Two main concentration regions are clearly visible: the stronger one, around the origin, with the highest peak and the lowest local variance, and the weaker one, in the bottom right region of the surface plot. Right: time evolution of mean positive and negative opinion. While the mean positive opinion quickly converges toward an equilibrium point, the negative one presents an increasing trend after a brief decreasing phase.

**Fig 9:**
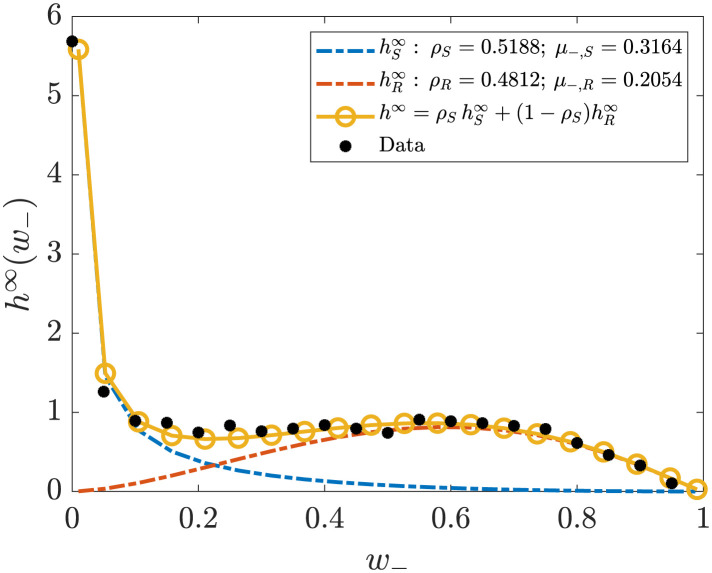
Fitting of equilibrium data: marginal of the negative opinion with a convex combination of the marginal distribution for susceptible and recovered individuals, respectively. The fitting parameters are shown and for ease of retrieval are also reported in Table 1.

The main peculiarity of the dataset is depicted in Fig 8: at the end of the evolution period, a significant concentration of people with strong negative opinion and essentially neutral positive opinion has formed. This is precisely the kind of clustering polarization that we mentioned in previous sections: here the dataset is showing a clear instance of bimodal distribution. Moreover, as the evolution of the positive and negative mean opinions shows, this bimodal distribution is the outcome of a polarizing trend across the population, which, interestingly, involves only the negative opinions. Let us focus on the marginal of the negative opinion at the final time snapshot. We make the *ansatz* that it can be approximated well by a suitable convex combination of two Beta distributions and we also assume that the distribution of data at the last time snapshot is the equilibrium of our dynamics. Then, we leverage our knowledge of the analytical expression for the steady state of the marginals (26) to solve the optimization problem

$$\begin{array}{c} \underset{\begin{array}{c} m_{-,S}^{\infty },\;m_{-,S}^{\infty },\\ \lambda_{-,S},\;\lambda_{-,R},\\ \sigma_{-,S},\;\sigma_{-,R},\\ \rho_{S}^{\infty } \end{array}}{\text{min}}\|\rho_{S}^{\infty }h_{S}^{\infty }(w_{-};m_{-,S}^{\infty },\frac{\lambda_{-,S}}{\sigma_{-,S}^{2}})+\left(1-\rho_{S}^{\infty }\right)h_{R}^{\infty }(w_{-};m_{-,R}^{\infty },\frac{\lambda_{-,R}}{\sigma_{-,R}^{2}})-{\underline{h}}^{\infty }\left(w_{-}\right){\|}_{2}, \end{array}$$ (27)

where ${\underline{h}}^{\infty }\left(\;\cdot \;\right)$ is the marginal distribution of the negative opinion extrapolated from the last recorded time snapshots, that is, we are fitting our parameters directly on the marginal distributions of the negative sentiments, which is the one with most features in our dataset. Problem (27) is solved via standard nonlinear least squares algorithm; we report in Table 1 the results we obtained from the fitting procedure and in Fig 9 the plot we obtain.

**Table 1:** Fitting parameters obtained as solution of minimization problems (27) and (28).

Opinion-related parameters						
*ρ*_*S*_	$m_{-,S}^{\infty }$	λ_−,*S*_	*σ*_−,*S*_	$m_{-,R}^{\infty }$	λ_−,*R*_	*σ*_−,*R*_
0.5188	0.0793	0.0475	0.3871	0.5574	0.0063	0.1756
	$m_{+,S}^{\infty }$	λ_+,*S*_	*σ*_+,*S*_	$m_{+,R}^{\infty }$	λ_+,*R*_	*σ*_+,*R*_
	0.0400	0.0412	0.4658	0.1375	0.0126	0.3228

The positive parameters were then established solving the analogous minimization problem

$$\begin{array}{c} \underset{\begin{array}{c} m_{+,S}^{\infty },\;m_{+,S}^{\infty },\\ \lambda_{+,S},\;\lambda_{+,R},\\ \sigma_{+,S},\;\sigma_{+,R},\\ \rho_{S}^{\infty } \end{array}}{\text{min}}\|\rho_{S}^{\infty }g_{S}^{\infty }(w_{-};m_{+,S}^{\infty },\frac{\lambda_{+,S}}{\sigma_{+,S}^{2}})+\left(1-\rho_{S}^{\infty }\right)g_{R}^{\infty }(w_{-};m_{+,R}^{\infty },\frac{\lambda_{+,R}}{\sigma_{+,R}^{2}})-{\underline{g}}^{\infty }\left(w_{-}\right){\|}_{2}, \end{array}$$ (28)

where this time ${\underline{g}}^{\infty }\left(\;\cdot \;\right)$ is the marginal distribution of the positive opinion from our dataset; the result is reported in Table 1.

Finally, once problem (27) is solved, we can also set the parameters responsible for the compartmental dynamics: we fixed *ζ* = *γ* = 1 to consider the average lifespan of a piece of fake news to be around 24 hours. The initial state we considered was *ρ*_*S*_(0) = 0.9, *ρ*_*E*_(0) = 0.05 and *ρ*_*I*_ = *ρ*_*R*_ = 0.025. We chose to have nonzero fractions of mass for the compartments of active spreaders and for the one of removed individuals to take into account exposure to fake news previous to the group-chat-related dynamics. Then, we used relation (23) along with our estimate of $\rho_{S}^{\infty }$ in Table 1 to determine the value of the basic reproduction number and therefore of *β* = 1.21.

### 5.2 Simulation results

The next section is devoted to compare the evolution provided by the model (16)–(19) and the one provided by the data. The model has been calibrated with the parameters in Table 1. For what concerns the epidemiological coefficients, they were chosen in order to achieve total masses at equilibrium that were compatible with the quantity *ρ*_*S*_ in Table 1, which gives the mass fractions of the two Beta distributions that concur to provide the steady state for the marginal negative density.

Computing the numerical evolution of the model requires a careful discretization of the system in order to keep high accuracy when describing the stationary solutions. To this aim we adopt the steady state preserving approach devised for Fokker-Planck equations in [69] by extending it to systems in the multidimensional case.

To this aim, system (16)–(19) was split both in time and opinion space. To describe the splitting, let us rewrite the system as follows

$$\partial_{t}\text{f}\left(\text{w},t\right)={\text{F}}_{+}\left[\text{f}\left(\text{w},t\right)\right]\left(w_{+},t\right)+{\text{F}}_{-}\left[\text{f}\left(\text{w},t\right)\right]\left(w_{-},t\right)+P\left[\text{f}\left(\text{w},t\right)\right]\left(\text{w},t\right),$$

where the bold operators are vector valued such as

$$\begin{array}{cc} {\text{F}}_{+}\left[\text{f}\left(\text{w},t\right)\right]\left(w_{+},t\right) & =[\frac{\partial }{\partial w_{+}}\left[(\lambda_{J}^{+}w_{+}-m_{+}\left(t\right))f_{J}\left(\text{w},t\right)\right]+\frac{\sigma_{+,J}^{2}}{2}\frac{\partial^{2}}{\partial w_{+}^{2}}(D{\left(w_{+}\right)}^{2}f_{J}\left(\text{w},t\right)){]}_{J},J\in C\\ {\text{F}}_{-}\left[\text{f}\left(\text{w},t\right)\right]\left(w_{-},t\right) & =[\frac{\partial }{\partial w_{-}}\left[(\lambda_{J}^{-}w_{-}-m_{-}\left(t\right))f_{J}\left(\text{w},t\right)\right]+\frac{\sigma_{-,J}^{2}}{2}\frac{\partial^{2}}{\partial w_{-}^{2}}(D{\left(w_{-}\right)}^{2}f_{J}\left(\text{w},t\right)){]}_{J},J\in C\\ \text{P}[\text{f}(\text{w},t)](\text{w},t) & =[-K\left(f_{S},f_{I}\right);K\left(f_{S},f_{I}\right)-\zeta f_{E};\zeta f_{E}-\gamma f_{I};\gamma f_{I}]\left(\text{w},t\right). \end{array}$$

Then, if we discretize the time domain with a time step of size Δ*t* > 0 and we denote by **f**^*n*^(**w**) an approximation of **f**(**w**, *n*Δ*t*), the (first-order) time splitting method consists in solving in the time interval [0, Δ*t*] the following sequence of problems

$$\begin{array}{c} \text{Evolve}\;\text{positive}\;\text{opinions}\;\Rightarrow \{\begin{array}{cc} \frac{\partial {\text{f}}^{†}}{\partial t} & ={\text{F}}_{+}\left[{\text{f}}^{†}\right],\\ {\text{f}}^{†}\left(\text{w},0\right) & ={\text{f}}^{n}\left(\text{w}\right),\; \end{array} \end{array}$$ (29)

$$\begin{array}{c} \text{Evolve}\;\text{negative}\;\text{opinions}\;\Rightarrow \{\begin{array}{cc} \frac{\partial {\text{f}}^{††}}{\partial t} & ={\text{F}}_{-}\left[{\text{f}}^{††}\right],\\ {\text{f}}^{††}\left(\text{w},0\right) & ={\text{f}}^{†}\left(\text{w},\Delta t\right), \end{array} \end{array}$$ (30)

$$\begin{array}{c} \text{Evolve}\;\text{fake-news}\;\text{spreading}\;\Rightarrow \{\begin{array}{cc} \frac{\partial {\text{f}}^{†††}}{\partial t} & =\text{P}[{\text{f}}^{†††}],\\ {\text{f}}^{†††}\left(\text{w},0\right) & ={\text{f}}^{††}\left(\text{w},2\Delta t\right), \end{array} \end{array}$$ (31)

and finally set **f**^*n*+1^(**w**) = **f**^†††^(**w**, Δ*t*). Higher order splitting can be constructed as well (see [69] and the references therein).

Each one-dimensional opinion direction was discretized using the structure-preserving scheme in [69] that for completeness is outlined below.

We want to solve a nonlinear Fokker-Planck equation of the form

$$\left\{ \begin{array}{cc} \partial_{t}f\left(w,t\right) & =\partial_{w}F\left[f\right]\left(w,t\right)\\ f(w,0) & =f_{0}\left(w\right), \end{array}\right.$$

where *t* ≥ 0, w∈Ω⊆R and F[·] is the so-called flux

$$F\left[f\right]\left(w,t\right)=\left(B\left[f\right]\left(w,t\right)+\partial_{w}D\left(w\right)\right)f\left(w,t\right)+D\left(w\right)\partial_{w}f\left(w,t\right),$$

with B[·] is a bounded operator describing aggregation dynamics and *D*(⋅) is a diffusion function. Now we introduce a uniform mesh *w*_*i*_ ∈ Ω, so that *w*_*i*+1_ − *w*_*i*_ = Δ*w*. We use the subscript notation $F_{i\pm 1/2}\left(t\right)$ to denote an approximation of the fluxes $F\left[f\right]\left(w_{i}\pm \Delta w/2,t\right)$. Let us consider the discretization

$$\frac{d}{dt}f_{i}\left(t\right)=\frac{F_{i+1/2}\left(t\right)-F_{i-1/2}\left(t\right)}{\Delta w},$$

where *f*_*i*_(*t*) approximates *f*(*w*_*i*_, *t*). The numerical flux $F_{i\pm 1/2}$ is calculated as

$$\left\{ \begin{array}{cc} F_{i+1/2} & ={\tilde{C}}_{i+1/2}{\tilde{f}}_{i+1/2}+D_{i+1/2}\frac{f_{i+1/2}-f_{i}}{\Delta w}\\ F_{i-1/2} & ={\tilde{C}}_{i-1/2}{\tilde{f}}_{i-1/2}+D_{i-1/2}\frac{f_{i}-f_{i-1/2}}{\Delta w}, \end{array}\right.$$

with the notation *D*_*i*±1/2_ = *D*(*w*_*i*±1/2_). Finally (omitting for simplicity the terms with subscript *i*−1/2, which are of analogous writing), we define

$$\begin{array}{c} {\tilde{f}}_{i+1/2}=\left(1-\delta_{i+1/2}\right)f_{i+1}+\delta_{i+1/2}f_{i},\\ \delta_{i+1/2}=\frac{1}{\lambda_{i+1/2}}+\frac{1}{1-\text{exp}(\lambda_{i+1/2})},\\ \lambda_{i+1/2}=\frac{\Delta w{\tilde{C}}_{i+1/2}}{D_{i+1/2}},\\ {\tilde{C}}_{i+1/2}=\frac{D_{i+1/2}}{\Delta w}\int_{w_{i}}^{w_{i+1}}\frac{B\left[f\right]\left(w,t\right)+\partial_{w}D\left(w\right)}{D(w)}\;dw. \end{array}$$

In the numerical simulations we used a coarse mesh of 20 points, whereas the time integration of the opinion evolution was computed with a semi-implicit scheme where the time step Δ*t* was chosen to be *O*(Δ*w*_+_), with Δ*w*_+_ = Δ*w*_−_ being the steps for the 2D opinion domain, whereas the epidemic exchange portion was integrated through a simple explicit method.

**Fig 10:**
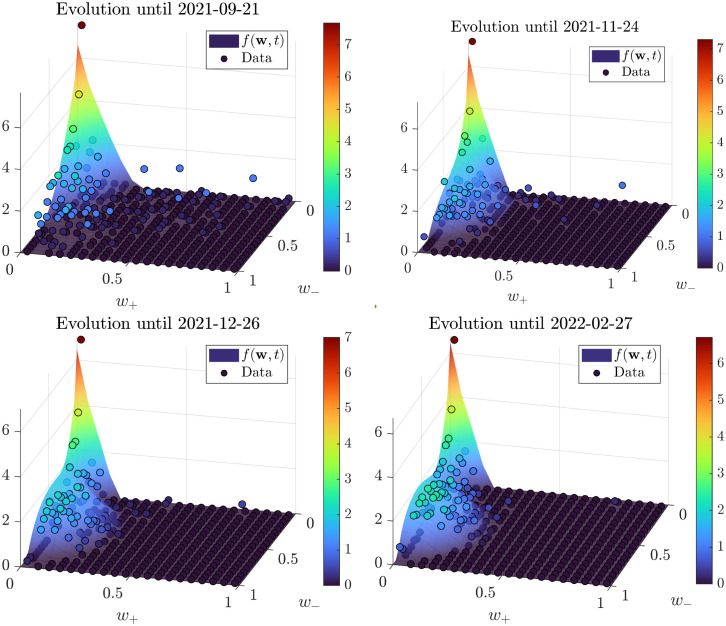
3D snapshots of dataset time series and model evolution. Again, base 2-logarithm of values is used for coloring. The warmer colors in the bottom left quadrant show the increasing polarization effect around negative opinions.

**Fig 11:**
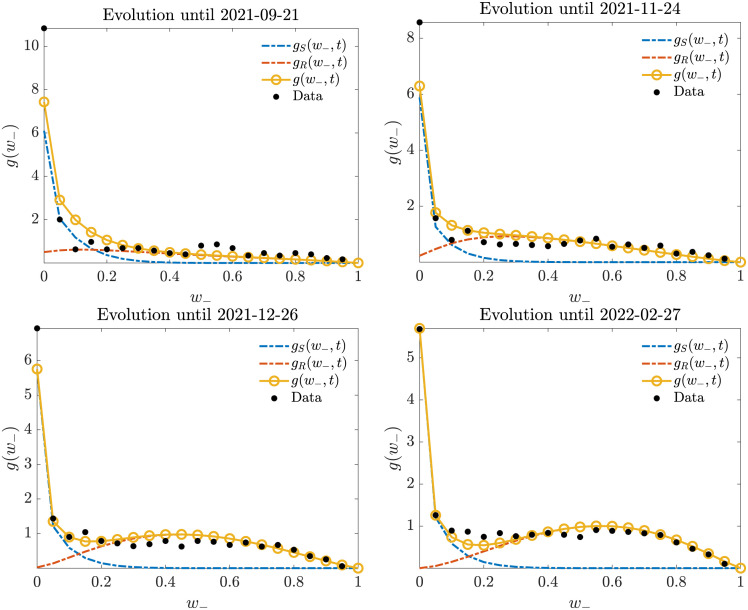
Evolution of the marginal density for the negative opinion: It is clear the emergence of a peak around the value of *w*_−_ = 0.6, i.e., a polarizing effect toward negative sentiments.

**Fig 12:**
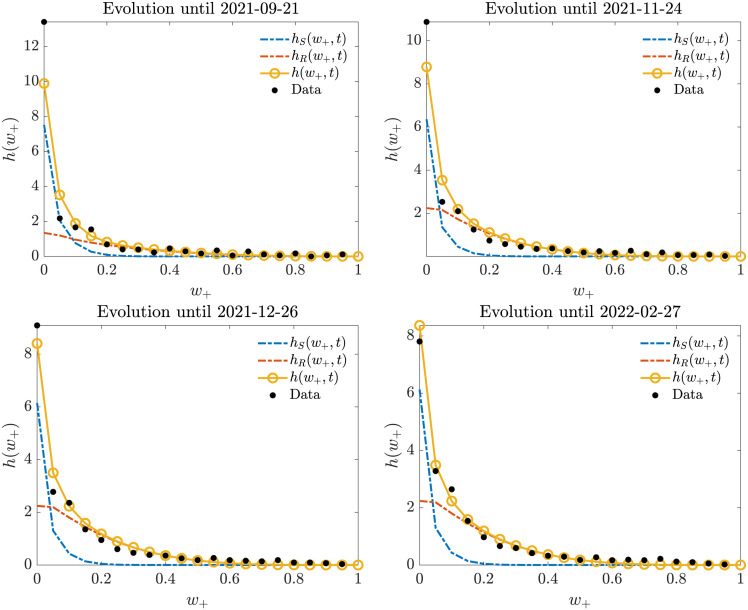
Evolution of the marginal density for the positive opinion: Even if the total distribution appears to be unimodal, it is still the sum of two distinct profiles.

In Fig 10 we report the comparison. Here, we can see that concentration around the origin starts very early, followed by a portion of data-points gathering towards a neighborhood of (0.25, 0.6) to form a peak later on. This polarization trend concerns only the negative opinions, as testified by the evolution of the marginal showed in Fig 11; while the mixture of equilibria for the positive marginal keeps substantially the same profile of unimodal decrease (Fig 12). Overall, we can see that the model is capable of correctly identifying the formation and evolution of both unimodal and bimodal trends happening at the same time in the two-dimensional evolution. To sum up, the model can accurately predict the polarization process towards negative extreme shown in our dataset.

## 6 Conclusion

Mathematical modeling of fake-news spreading is a particularly timely and challenging topic, involving numerous areas of research with strong social impacts. In this paper we focused on opinion formation processes within closed communities in presence of spreaders of misinformation. Inspired by a real case study from social data using NLP techniques, we presented a data-driven model based on vector stochastic differential equations. Then, in order to analyze the model and compute analytically the stationary solutions for its spatial marginals, we considered its mean-field approximation in the form of a system of Fokker-Planck equations, where the dissemination of fake news was carried on through a compartmental approach. Finally, we compared the evolution of the model computed numerically with the one of the dataset time series extract using sentiment analysis. Our results show a good agreement between them, allowing us to observe the formation of bimodal distributions indicating the polarization of opinions toward very negative sentiments as manifested in the real data. We emphasize that the present model, due to its generality, naturally lends itself to many other areas of application in relation to the analysis of fake-news dissemination using NLP techniques in different contexts.

## Supporting information

S1 Data (CSV)
